# Asthma prevalence among 16- to 18-year-old adolescents in Saudi Arabia using the ISAAC questionnaire

**DOI:** 10.1186/1471-2458-12-239

**Published:** 2012-03-26

**Authors:** Mohammed O Al Ghobain, Mohamad S Al-Hajjaj, Mohamad S Al Moamary

**Affiliations:** 1 Department of Medicine, College of Medicine, King Saud bin Abdulaziz University for Health Sciences, Riyadh, Saudi Arabia; 2 Department of Medicine, College of Medicine, King Saud University, Riyadh, Saudi Arabia; 3 College of Medicine, King Saud bin Abdulaziz University for Health Sciences, P.O. Box 90068, Riyadh 11321, Saudi Arabia

## Abstract

**Background:**

Most of the studies investigating the prevalence of asthma in various countries have focused on children below the age of 15 years or adults above the age of 18 years. There is limited knowledge concerning the prevalence of asthma in 16- to 18-year-old adolescents. Our objective was to study the prevalence of asthma and associated symptoms in 16- to 18-year-old adolescents in Saudi Arabia.

**Methods:**

A cross-sectional study was conducted in secondary (high) schools in the city of Riyadh utilizing the International Study of Asthma and Allergies in Children (ISAAC) questionnaire tool.

**Results:**

Out of 3073 students (1504 boys and 1569 girls), the prevalence of lifetime wheeze, wheeze during the past 12 months and physician-diagnosed asthma was 25.3%, 18.5% and 19.6%, respectively. The prevalence of exercise-induced wheezing and night coughing in the past 12 months was 20.2% and 25.7%, respectively. The prevalence of rhinitis symptoms in students with lifetime wheeze, physician-diagnosed asthma and exercise-induced wheeze was 61.1%, 59.9% and 57.4%, respectively. Rhinitis symptoms were significantly associated with lifetime wheeze (OR = 2.5, *p *value < 0.001), physician-diagnosed asthma (OR = 2.2, *p *< 0.001), and exercise-induced wheeze (OR = 1.9, *p *value < 0.001).

**Conclusions:**

The prevalence of asthma and associated symptoms in 16- to 18-year-old adolescents in Saudi Arabia is high, although it is within range of reported prevalence rates from various parts of the world.

## Background

Asthma is one of the most common chronic diseases in children and adults. The prevalence of asthma has increased in developed and developing countries over the last three decades. Standardized research instruments, such as the International Study of Asthma and Allergies in Children (ISAAC) questionnaire for children and the European Community Respiratory Health Survey (ECRHS) for adults, have been introduced to gain better insight into the worldwide prevalence of asthma and other allergic diseases [1,2]. The ISAAC questionnaire was developed to determine the prevalence of asthma, rhinitis and eczema in various locations. The main objective of the ISAAC was to facilitate comparisons between countries and to assess future tendencies regarding the prevalence of asthma and other allergic diseases [1]. The ISAAC protocol was developed to target populations of at least 3000 children ages 13 to 14 years old and 6 to 7 years old per center. The Phase One of the ISAAC study, which included 156 centers in 56 countries and a total of 721,601 participants, reported large variations in the prevalence of asthma symptoms in many regions worldwide [1]. Phase three of the ISAAC study occurred between 2000 and 2003 and included 233 centers in 97 countries, with a total of 798,685 participants aged 13 to 14 years, and revealed wide variations in the prevalence of asthma in this age group worldwide [3]. The highest asthma rates have been reported in affluent countries, such as the United Kingdom, New Zealand, and Australia, whereas the lowest rates have been reported in India and Indonesia. The severity of asthma symptoms has been shown to be greater in less affluent countries, such as Costa Rica.

The prevalence of asthma in Saudi Arabia has been investigated in several previous studies. Al Frayh et al. conducted epidemiological studies in Saudi Arabia in 1986 and 1995 and showed that the prevalence of asthma in comparable populations increased from 8% to 23%, respectively [4]. Hijazi et al. investigated the prevalence of asthma in 1,020 urban and 424 rural children and found that the prevalence of asthma was 13.9% and 8%, respectively [5]. Al-Dawood et al. reported that the prevalence of physician-diagnosed asthma in school-age boys was 8% [6]. Similarly, Alshehri et al. found that the prevalence of asthma in school-age boys was 9% [7].

Many epidemiological studies investigating asthma in countries worldwide have focused on children below the age of 15 (ISAAC protocol) or on adult populations (ECRHS protocol). To our knowledge, only a limited number of studies have investigated the prevalence of asthma in adolescents aged 16 to 18 years. There is a gap in the knowledge concerning the prevalence of asthma in boys and girls in the 16- to 18-year old age range. This study was designed to bridge the data gap in this under-investigated age group. Our objectives were to study the prevalence of asthma using the ISAAC questionnaire and to characterize and measure the frequency of asthma symptoms in male and female secondary (high) school students aged 16 to 18 years in Riyadh, Saudi Arabia. This study is part of larger national survey conducted by the Saudi thoracic society to describe the prevalence and characteristics of asthma, rhinitis and smoking among high school students in Riyadh, Saudi Arabia.

## Methods

A cross-sectional survey using the ISAAC questionnaire was administered to secondary (high) school students (16 to 18 years old) in Riyadh, Saudi Arabia. There are 160 secondary schools for boys (72 private schools) and 245 secondary schools for girls (113 private schools) in Riyadh. The total number of Saudi secondary school students in Riyadh during the 2009-2010 academic year was 161,223 students, including 83,056 boys (51.5%) and 78,167 girls (48.5%). Schools and students were selected using a two-stage cluster sample. In the first sampling stage, a random selection method was used to select 46 secondary schools in Riyadh from a list of 405 schools, for a total of 19,834 Saudi students (9,771 boys and 10,063 girls). We divided Riyadh into four districts (northern, eastern, southern, and western) based on the proximity to two major highways. A total of 15% of the schools for boys and 10% of the schools for girls were selected based on proportional probability in each district. Fourteen schools were selected from the eastern district (7 schools for boys and 8 schools for girls), 12 schools from the northern district (6 schools for boys and 6 schools for girls), 10 schools from the western district (5 schools for boys and 4 schools for girls), and 10 schools from the southern district (5 schools for boys and 5 schools for girls). Three classes, or one per grade level, were selected from each school during the second sampling stage for a total of 138 classes. Each class was considered to be a cluster, and all of the students in the selected classes constituted the target group.

The participants were interviewed by trained medical students and completed questionnaires in the classroom under the supervision of the medical students. The medical students received a half-day training session that included an overview of the study and its methodology and were taught to avoid explanations that could interfere with the participants' answers. The following variables were collected from the ISAAC questionnaire: lifetime wheeze (if wheezing had ever occurred), wheeze in the past 12 months, number of wheezing attacks in the past 12 months, sleep disturbances due to wheeze, speech-limiting wheeze, physician-diagnosed asthma (if asthma had ever been diagnosed by physician), exercise-induced wheeze, and night coughing in the past 12 months.

The survey was conducted with permission from the Ministry of Education. We received approval from the Research and Ethics Committee of the Saudi Thoracic Society. Verbal consent was provided by the students after the purpose of the study was explained. Students were assured that the surveys would be anonymous and that the data would be kept confidential during handling and storage.

The data were analyzed using a statistical software tool (PASW 18.0). All variables were summarized and reported using descriptive statistics. An odds ratios (OR) with a corresponding 95% confidence interval (CI) was calculated for the risk factors (e.g., type of school, rhinitis symptoms and hay fever) that exhibited a significant association with prevalence. A *P*-value of 0.05 or less was considered significant.

## Results

A total of 3400 questionnaires were distributed to the students, and 117 students refused to participate. The overall participation rate was 96.5%. Because 210 questionnaires were excluded due to incomplete data, the final total sample size was 3,073 students. Boys completed 1,504 questionnaires (48.9%), and girls completed 1,569 (51.1%). The baseline characteristics of the study population are shown in Table 1.

**Table 1:** Baseline characteristics of the study group


	**Boys** **No. (%)**	**Girls** **No. (%)**	**All** **No. (%)**
	
**Total students**	1504 (48.9%)	1569 (51.1%)	3073 (100.0%)
**Age**			
Grade 1	510 (33.9%)	504 (32.1%)	1014 (33.0%)
Grade 2	492 (32.7%)	533 (34.0%)	1025 (33.4%)
Grade 3	502 (33.4%)	532 (33.9%)	1034 (33.6%)
**Type of school**			
Government	677 (45.0%)	817 (52.1%)	1494 (48.6%)
Private	827 (55.0%)	752 (47.9%)	1579 (51.4%)
**School area**			
North	480 (31.9%)	376 (24.0%)	856 (27.9%)
East	366 (24.3%)	566 (36.1%)	932 (30.3%)
South	308 (20.5%)	335 (21.4%)	643 (20.9%)
West	350 (23.3%)	292 (18.6%)	642 (20.9%)

The prevalence of lifetime wheeze, wheeze during the past 12 months, and physician-diagnosed asthma was 25.3%, 18.5% and 19.6%, respectively. The prevalence of exercise-induced wheeze and night cough in the past 12 months was 20.2% and 25.7%, respectively (Table 2). There was no significant difference in lifetime wheeze between boys and girls (26.5% vs. 24.3%) (*p *= 0.165). However, the boys reported more wheezing symptoms over the past 12 months (*p *< .016), physician-diagnosed asthma (*p *< 0.009), exercise-induced wheeze (*p *≤ 0.001) and night cough (*p *< 0.001) compared to the girls.

**Table 2:** The prevalence of wheeze and associated symptoms in the study group

	**Boys**	**Girls**	**All**	
	
	**No. (%)**	**No. (%)**	**No. (%)**	***P *value**
	
**Lifetime wheeze**	398 (26.5%)	381 (24.3%)	779 (25.3%)	0.165
**Wheeze in the past 12 months**	305 (20.3%)	265 (16.9%)	570 (18.5%)	0.016
**Physician-diagnosed asthma**	324 (21.5%)	279 (17.8%)	603 (19.6%)	< 0.009
**Number of wheezing attacks in the** **past 12 months**				
1-3	270 (64.9)	227 (70)	499 (67.3)	
4-12	81 (19.5)	67 (20.6)	148 (19.9)	0.003
> 12	65 (15.6)	30 (9.3)	95 (12.8)	
**Sleep disturbances due to wheeze**				
≤ Nights/week	444 (88.7%)	425 (90.0%)	869 (89.3%)	
> Nights/week	57 (11.4%)	47 (10.0%)	104 (10.7%)	0.715
**Speech-limiting wheeze**	120 (28.5%)	139 (42.5%)	259 (34.6%)	< 0.001
**Exercise-induced wheeze**	340 (22.6%)	282 (18.0%)	622 (20.2%)	< 0.001
**Night cough in the past 12 months**	301 (38.1%)	488 (31.1%)	789 (25.7%)	< 0.001

In assessing the severity of asthma symptoms, the majority of the students (67.3%) experienced fewer than 3 wheezing attacks in the past 12 months (Table 2). A total of 19.9% students had four or more wheezing attacks, which was a significantly higher parameter in the boys than the girls (*p *< 0.003). Regarding sleep disturbances, 10.7% were awakened more than one night per week due to wheezing. The prevalence of speech-limiting wheeze was 34.6%. The associations between lifetime wheeze, physician-diagnosed asthma and exercise-induced wheeze at the government schools were 48.5%, 46.6%, and 47.9%, respectively. These results did not show significant differences between the type of the school and lifetime wheeze, physician-diagnosed asthma and exercise-induced wheeze in both genders, with p values of 0.952, 0.269 and 0.693, respectively.

The prevalence of rhinitis symptoms in students with lifetime wheeze, physician-diagnosed asthma and exercise-induced wheeze was 61.1%, 59.9% and 57.4%, respectively (Table 3). Rhinitis symptoms were strongly linked with lifetime wheeze (OR = 2.5, *p *< .001), physician-diagnosed asthma (OR = 2.2, *p *< .001), and exercise-induced wheeze (OR = 1.9, *p *< .001). The prevalence of hay fever in students with lifetime wheeze, physician-diagnosed asthma and exercise-induced wheeze was 33.6%, 34.3% and 32.6%, respectively. Hay fever was strongly linked with lifetime wheeze (OR = 2.4, *p *< .001), physician-diagnosed asthma (OR = 2.3, *p *< .001), and exercise-induced wheeze (OR = 2.1, *p *< .001).

**Table 3:** Asthma symptoms in relation to rhinitis symptoms and hay fever

	**Rhinitis symptoms**				**Hay fever**			
	
	**Prevalence** **No. (%)**	***P *value**	**Odds Ratio**	**Confidence interval**	**Prevalence** **No. (%)**	***P *value**	**Odds Ratio**	**Confidence interval**
	
**Lifetime wheeze**	476 (61.1%)	< .001	2.5	2.1-3.0	262 (33.6%)	< .001	2.4	2.0-2.9
**Physician-diagnosed asthma**	361 (59.9%)	< .001	2.2	1.8-2.6	207 (34.3%)	< .001	2.3	1.9, 2.8
**Exercise-induced wheeze**	357 (57.4%)	< .001	1.9	1.6, 2.3	203 (32.6%)	< .001	2.1	1.7, 2.6

## Discussion and Conclusions

This study has established the prevalence of asthma symptoms in students aged 16 to 18 years in Saudi Arabia and the relationship between asthma and rhinitis symptoms and hay fever. The prevalence of lifetime wheeze, wheeze during the past 12 months and physician-diagnosed asthma was 25.3%, 18.5% and 19.6%, respectively. The prevalence of exercise-induced wheezing and night coughing during the past 12 months was 20.2% and 25.7%, respectively. This study is unique in that it is the first evaluation to utilize the ISAAC questionnaire to address the prevalence of asthma among high school students aged 16 to 18 years in Saudi Arabia. Many of the previous asthma prevalence studies in Saudi Arabia and other Gulf countries were primarily conducted in children below the age of 15 years using either the ISAAC questionnaire or other research tools to screen for asthma [4,6,8-13]. The overall prevalence of asthma in Saudi children has been reported to range from 8% to 25% based on studies conducted over the past 3 decades [4,6,8,9]. The highest prevalence of physician-diagnosed bronchial asthma in Saudi Arabia was reported to be 25% in 2004, but the primary aim of that study was to estimate the prevalence of allergic rhinitis in children [8]. Epidemiological studies in Saudi Arabia revealed an increasing prevalence of asthma the past three decades that may be attributed to rapid lifestyle changes related to the modernization of Saudi society, changes in dietary habits, and exposure to environmental factors, such as indoor allergens, dust, sand storms and tobacco. Additionally, this high prevalence of asthma could be attributed to an increase in asthma awareness in the general population and among healthcare workers.

Our study showed that boys reported more wheezing symptoms during the past 12 months, physician-diagnosed asthma, exercise-induced wheeze, and night coughing compared to girls. This may be related to physiologic differences between the genders. However, cultural differences could influence behaviors that may lead to over-reporting or under-reporting of symptoms. Our findings did not show a significant association between the prevalence of asthma and governmental or private schools, which may be explained by consistencies in socioeconomic status and access to the healthcare system. Other studies have reported different observations between public and private schools [14].

In other Gulf countries, the prevalence of childhood physician-diagnosed asthma was reported to be 16.8% in Kuwait (2000), 13% in the United Arab Emirates (2000), 19.8% in Qatar (2006) and 10.6% in Oman (2008) [10-13]. Phase one of the ISAAC study revealed that the prevalence of wheezing in the past 12 months ranged from 4.1% to 32.1% in 6- to 7-year-old children and 2.1% to 32.2% in 13- to 14-year-old children worldwide. In Phase Three of the ISAAC study, the prevalence of wheezing in the past 12 months was between 2.4% and 37.6% in 6- to 7-year-old children and 2.1% to 32.2% in 13- to 14-year-old children. The prevalence of physician-diagnosed asthma ranged from 3.4% to 29.2% in the 13- to 14-year-old age group [3].

Our findings revealed that the prevalence of rhinitis symptoms and hay fever were high and were strongly linked to subjects with asthma. This result is consistent with previous results reported in the literature. Asthma and rhinitis often represent a spectrum of the same disease (the one airway hypothesis) [15]. In one study, rhinitis occurred in 75 to 90% of adult subjects with allergic asthma and in 80% of adults with non-allergic asthma [16]. In another study, rhinitis occurred in 40 to 75% of all adults and children with asthma [17]. In Britain, 53% of the boys and 61% of the girls with asthma had rhinitis symptoms, [18] and in Greece, 69% of children with asthma had rhinitis symptoms [19]. The association between asthma and rhinitis is related to the neural nasal-bronchial interaction, disturbances in the warming and humidification functions of the nasal mucosa, drainage of irritants and inflammatory materials into the lungs and the presence of similar cellular infiltrates and pro-inflammatory mediators in the upper and lower airways.

In conclusion, the prevalence of asthma and asthma-related symptoms is high among 16- to 18-year-old adolescents in Saudi Arabia, and the symptoms are more common in boys than in girls. Asthma and asthma-related symptoms are also associated with a high rate of rhinitis symptoms and hay fever. The high prevalence of asthma in Saudi Arabia is within the reported prevalence ranges from many other parts of the world.

## Competing interests

The authors declare that they have no competing interests.

## Authors' contributions

MA-G, primary author, study design, analysis, and writing. MA-H, study design, approvals, and reviewing the manuscript. MA-M, study design, implementation of the study, and writing. All authors read and approved the final manuscript.

## Pre-publication history

The pre-publication history for this paper can be accessed here:

http://www.biomedcentral.com/1471-2458/12/239/prepub
